# Going green? Ex-post valuation of a multipurpose water infrastructure in Northern Italy

**DOI:** 10.1016/j.ecoser.2017.07.015

**Published:** 2017-10

**Authors:** Arnaud Reynaud, Denis Lanzanova, Camino Liquete, Bruna Grizzetti

**Affiliations:** a Toulouse School of Economics, INRA, University of Toulouse Capitole, Toulouse, France; b Center for Development Research (ZEF), University of Bonn, Bonn, Germany; c European Commission, Joint Research Centre (JRC), Directorate D – Sustainable Resources, Ispra, Italy

**Keywords:** Ecosystem services, Green infrastructure, Nature-based solution, Economics, Contingent valuation

## Abstract

A contingent valuation approach is used to estimate how households value different multipurpose infrastructures (conventional or green) for managing flood risk and water pollution. As a case study we consider the Gorla Maggiore water park located in the Lombardy Region, in Northern Italy. The park is a neo-ecosystem including an infrastructure to treat waste water and store excess rain water, built in 2011 on the shore of the Olona River in an area previously used for poplar plantation. This park is the first one of this type built in Italy. A novel aspect of our research is that it not only considers the values people hold for different water ecosystem services (pollution removal, recreative use, wildlife support, flood risk reduction), but also their preferences for how those outcomes are achieved (through conventional or green infrastructures). The results indicate that the type of infrastructure delivering the ecosystem services does have an impact on individuals’ preferences for freshwater ecosystem services. Households are willing to pay from 6.3 to 7.1 euros per year for a green infrastructure (compared to a conventional one), with a premium up to 16.5 euros for a surrounding made of a park. By considering the type of infrastructure within the choice model, we gain a richer understanding of the relationship between social welfare and freshwater ecosystem services.

## Highlights

• We use a CV approach to estimate how households value a green infrastructure.
• As a case study we consider the Gorla Maggiore water park (Northern Italy).
• We find a positive willingness to pay for the green infrastructure (16.5 euros/hh/y).
• Building of the green infrastructure can be rationalized based on a cost-benefit criterion.

## 1 Introduction

Green infrastructures “comprise of all natural, semi-natural and artificial networks of multifunctional ecological systems within, around and between urban areas, at all spatial scales” (Tzoulas et al., 2007). Green infrastructures then refer to the living network of green spaces, water and other environmental features in both urban and rural areas. This concept is often used in an urban context to cover benefits provided by trees, parks, gardens, woodlands, rivers and wetlands. There is a long list of potential benefits provided by green infrastructures that the European Environmental Agency (2011) reviewed and classified in ten broad topics: biodiversity/species protection, climate change adaptation, climate change mitigation, water management, food production and security, recreation well-being and health, land values, culture and communities. Recently, the European Commission (2013) has defined green infrastructure as “a strategically planned network of natural and semi-natural areas with other environmental features designed and managed to deliver a wide range of ecosystem services”.

A large literature identifying the benefits to be expected from green infrastructure has developed in the last decades. Among others, Tzoulas et al. (2007) have reviewed the literature on green infrastructure in relationship with ecosystem health, human health and human well-being. Wang et al. (2014) have summarized the literature from different disciplines to synthesize the knowledge on the effects of green infrastructures on the indoor environment and human comfort in urban areas.

Despite the abundant literature in urban planning (Gill et al., 2007, Pugh et al., 2012, Ellis, 2013), published economic analyses focusing on green infrastructures remain still quite limited. Jim and Chen (2006) have used a contingent valuation method to evaluate the recreational amenities of urban green spaces in Guangzhou, China. Using the same valuation approach, López-Mosquera and Sánchez (2011) have shown that a higher environmental and social awareness is associated with a higher willingness to pay for the Monte de San Pedro Natural Park, a peri-urban green space located in Coruña (Spain). In the same vein, Mell et al. (2013) value the development of green infrastructure investments (trees) in the urban core of Manchester, UK. Benefits and costs of street trees have been also assessed in Lisbon, Portugal (Soares et al., 2011) and in Portland, US where it has been shown that the number of street trees fronting the property and crown area within 30.5 m of a house positively influence sales price (Donovan and Butry, 2010). Wilker and Rusche (2014) have used a contingent valuation approach to value different types of green infrastructures in Esslingen, Germany. They analyze how the elicited willingness to pay can be integrated in regional planning policies. Use of economic valuation to create public support for green infrastructures is also discussed in Vandermeulen et al. (2011). The perspective of Baptiste et al. (2015) is a little bit different since the authors focus on the factors that influence the public’s willingness to implement green infrastructures on private properties.

Our paper aims at contributing to the literature providing economic values for green infrastructures. Our specific focus is on green infrastructures dedicated to water pollution removal and flood risk management. As a case study we consider the Gorla Maggiore water park located in the Lombardy Region, in Northern Italy. This park is a neo-ecosystem including a green infrastructure to treat waste water and store excess rain water, built in 2011 on the shore of the Olona River in an area previously used for poplar plantation. The Gorla Maggiore park is the first one of this type built in Italy. We contribute to the literature on valuation of green infrastructures in three different ways. First, our research considers the values people hold for different water ecosystem services (pollution removal, recreative use, biodiversity, flood risk reduction) and also their preferences for how those outcomes are achieved (through conventional or green infrastructures). By considering the type of infrastructure within the choice model, we gain a richer understanding of the relationship between social welfare and freshwater ecosystem services. Second, we propose the first application of the *attribute-based* contingent valuation approach developed by Moore et al. (2011) to the context of ecosystem services. Third, our valuation study has been conducted ex-post, a few years after the construction of the Gorla Maggiore water park. Since people have already benefited from the services provided by this park, this might reduce the hypothetical concerns usually attributed to using a stated preference approach.

The remaining of the paper is organized as follows. Section 2 describes our case study in Italy and Section 3 is devoted to presenting the design of the contingent valuation survey and its administration. The results of the econometric model are reported in Section 4, and Section 5 concludes the paper.

## 2 The Gorla Maggiore water park

**Fig. 1:**
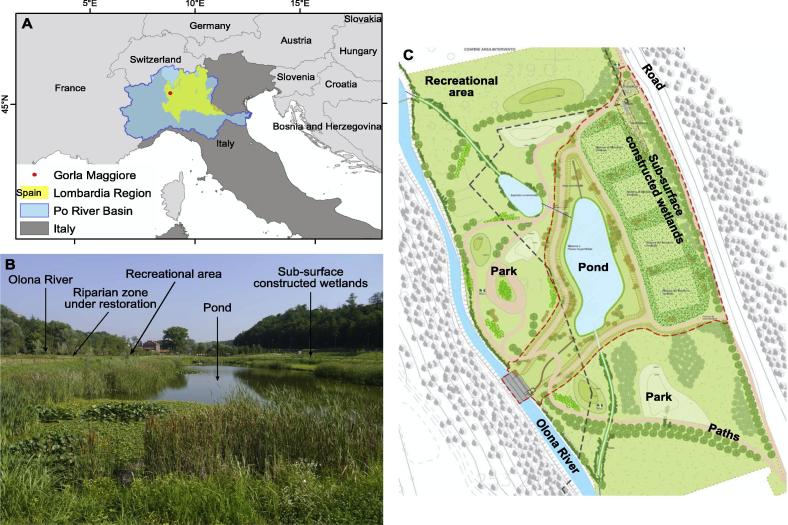
Location and characteristics of the Gorla Maggiore water park.

The municipality of Gorla Maggiore (located in the Lombardy Region, in Northern Italy, Fig. 1) operates a typical combined sewer system designed to collect rainwater runoff, domestic sewage, and industrial wastewater in the same pipe network. Most of the time, the combined sewer system transports all the sewage to the wastewater treatment plant of Olgiate Olona (located about 7 km downstream Gorla Maggiore), where it is treated and then discharged in the Olona River. During periods of heavy rainfall, however, the water volume can exceed the capacity of the combined sewer system and creates an overflow that is discharged directly into the Olona River. Overflows contain not only storm water but also untreated human and industrial waste, toxic materials and debris, and can contribute to local flooding. These events are frequent in Gorla Maggiore where just between March and August 2014, 70 overflows episodes were registered (Masi et al., 2015). To address this issue, the Lombardy Regional Authority has reinforced a law (R.R.n.3 from 24 March 2006), compliant with the EU Water Framework Directive, that forces all municipalities to treat their combined sewer overflow. Constructed wetlands are starting to be considered as an eco-suitable technology to treat combined sewer overflows Meyer et al. (2012). In 2011–2012, an innovative green infrastructure was built in Gorla Maggiore (the first one of this type in Italy) that addresses the issue of water pollution and flood control.

The green infrastructure consists in a set of constructed wetlands, surrounded by a park (Fig. 1). All together the constructed wetlands and the surrounding park form the Gorla water park. This neo-ecosystem was built on the shore of the Olona River in an area previously used for poplar plantation. The Gorla water park has been developed under the sponsorship of the Lombardy Regional Authority and co-funding by Fondazione Cariplo, and it has been designed by IRIDRA, an engineering firm founded in 1998 by a multidisciplinary group of professionals (biology, chemistry, engineering) with experience in sustainable water management. IRIDRA’s field of excellence is the design of constructed wetlands for wastewater treatment. The whole area surface of the Gorla water park is about 6.5 ha. It includes (a) a pollutant removal area (1 ha) composed of a grid, a sedimentation tank and four vertical sub-surface flow constructed wetlands; (b) a multipurpose area (1 ha) with a surface flow constructed wetland (the pond in Fig. 1) with multiple roles, such as pollution retention (secondary and tertiary treatment), buffer tank for flood events, maintenance of biodiversity and recreational area; and (c) a recreational park (4.5 ha) with restored riparian trees, green open space, walking and cycling paths and some services (e.g. picnic table, toilets, bar) maintained by a voluntary association ( http://www.calimali.org/).

The Gorla water park is a multi-purpose green infrastructure since it also includes a leisure and recreational area which is dedicated to a wide range of activities including educational activities, biking, running, picnicking, animal watching. In addition, several educational services related to the presence of fauna are available on the site (water birds and small amphibians) and advertised by informational panels. Flora is highlighted, especially for the plants (emerged and floated leaved macrophytes) involved in the water purification processes. The accessibility is excellent (600 meters from the town of Gorla Maggiore through a foot path). The park has been particularly well designed for educative activities with a dedicated small pond where frogs can be very easily observed, and with many informational panels.

To summarize, the Gorla Maggiore water park has been designed to provide four different types of water ecosystem services:

• Pollution control (reduction of the pollution load discharged into Olona River by a combined sewer overflow),
• Flood prevention (storage of rainwater and regulation of flow discharge to the river),
• Recreational use (use of the park by the local population),
• Biodiversity or wildlife support (provide habitats for birds, macroinvertebrates or amphibians species, among others).

This infrastructure showcases the capacity of human to mimic nature’s functions. Purely “natural” services such as pollution or flood control have been enhanced by the use of technologies and large inputs of manufactured capital. Recreation also results from a strong interaction between capital and ecosystem processes. In that respect, the Gorla Maggiore park is an example of ecosystem service co-production, as defined by Lele et al. (2013). The changes in biophysical variables (e.g. water quantity or amount of treated water) and improved well-being (e.g. better affect from nature experience) are the result of physical and cognitive co-production (Palomo et al., 2016).

## 3 The contingent valuation survey

A wide range of economic valuation techniques could have been used to value the ecosystem services provided by the Gorla Maggiore water park. Due to its high level of flexibility, our preferred valuation method would have been a discrete choice experiment. However, due to the mode of administration of the survey (mail) and the fact that the valuation exercise has been conducted ex-post (i.e. a few years after the construction of the park), we have chosen to use a contingent valuation (CV) approach. In the absence of a market price, it provides a direct method for estimating the monetary value of an environmental resource (Mitchell and Carson, 1989). A recent application of the CV to the valuation of water ecosystem services is Pinto et al. (2016).

Our CV approach is not standard in two aspects. First, in our work, respondents will be asked to answer sequentially four CV questions. In each case, they will have to compare the ecosystem services provided in a reference scenario (the situation which used to prevail before the construction of the water park) with those derived from an alternative infrastructure which was feasible when the park was built. Second, each infrastructure will be described by a set of attributes. This allows us to examine the tradeoffs that people are willing to make between ecosystem services provided by the different infrastructures. But rather than varying the attribute levels across infrastructures according to a specific design (as it is usual done when using discrete choice experiment), in our case the combination of attributes for a given infrastructure is chosen to represent a feasible infrastructure that was really considered by policy-makers at the time at which the water park has been built. We have implemented an *attribute-based* CV approach, in the spirit of what has been done by Moore et al. (2011) in the context of forest protection programs. However, even if we follow their approach, ours differs in three dimensions. First, each program is here characterized by a larger number of attributes (four against two). Second, our attributes are directly related to the provision of ecosystem services, which is not the case in their work. Third, the context of our study is also different since we focus on delivery of ecosystem services whereas the main issue they addressed was conservation of sites.

### 3.1 Development of the survey

The survey has been developed by an interdisciplinary team including ecologists, biologists, hydrologists and environmental economists. The starting point for developing the survey has been a field trip organized in July 2014 in the Gorla water park. We conducted there different scientific activities including sampling in the pond for macroinvertebrates, sampling in the river for macroinvertebrates, evaluating plant biodiversity in the artificial wetland and identifying the eco-recreational potential of the area. A first English version of the survey was then designed following this field trip and tested internally at the Joint Research Centre of the European Commission (by four scientists from different disciplines). Some parts of the survey were then amended and the survey was translated in Italian by an Italian native speaker. This second version of the survey was then submitted for comments and discussions to some representatives of the municipality of Gorla Maggiore and to the engineering company who designed the Gorla Maggiore park. By accounting for these comments (in particular those related to the payment vehicle to be used) we ended with the final version of the CV survey consisting of three sections. In the first section, we measure how often individuals have visited the Gorla Maggiore park in the last 12 months. We also collect information regarding the type of recreational activities undertaken by individuals when visiting the park. The second section is the main CV part of the survey. In the third section, we collect some basic socioeconomic information on respondents and identify protest answers.

### 3.2 Contingent valuation section of the questionnaire

**Fig. 2:**
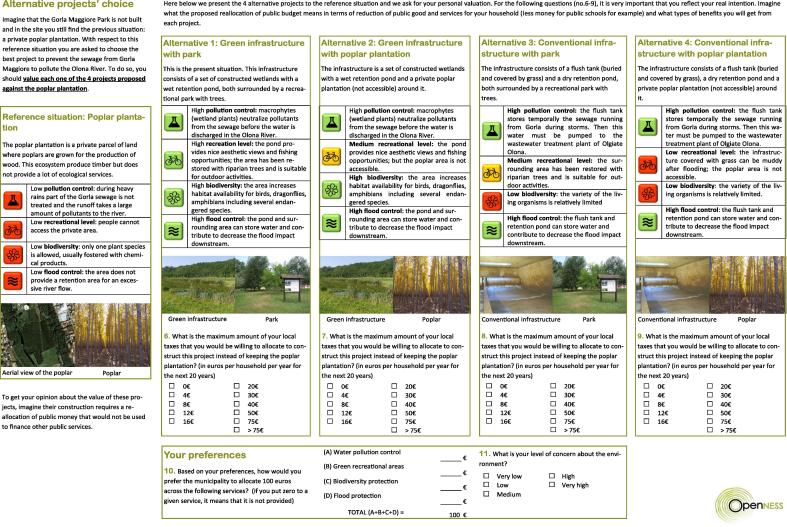
Contingent valuation section of the questionnaire.

The survey focuses on the willingness to pay (WTP) for several contingent valuation scenarios (green or conventional infrastructure providing different environmental benefits), to be compared to a reference scenario (Fig. 2).

*Reference scenario*. We first describe a reference situation in which the whole area is covered by a private poplar plantation. This situation before the construction of the Gorla Maggiore park is defined as being the *reference scenario* and is described in the questionnaire as:

*“Imagine that the Gorla Maggiore Park is not built and in the site you still find the previous situation: A private poplar plantation […]. The poplar plantation is a private parcel of land where poplars are grown for the production of wood. This ecosystem produces timber but does not provide a lot of ecological services.”*

Since a crucial issue is the good understanding by respondents of the characteristics of the reference scenario, we describe explicitly in the questionnaire the level of ecosystem services provided in terms of pollution reduction, recreational activities, biodiversity and flood protection associated to this scenario. As it can be seen in Fig. 2, the reference scenario corresponds to a situation characterized by a low pollution control, low recreational levels, a low biodiversity and a low flood control. Both the phrasing and the quantification of ecosystem services associated to the reference scenario (and also to the four alternative scenarios) have been discussed and validated by natural scientists and by IRIDRA, the engineering firm which was responsible for the design and the construction of the Gorla Maggiore park.

The verbal description to quantify ecosystem services associated to the reference scenario was accompanied by visual aids for facilitating a full understanding of the valuation scenarios, see Fig. 2. As Mitchell (2002) points out, visual aids play a vital role both in illustrating the verbal information and in holding respondents’ attention during the presentation of scenarios. We have used two types of visual aids. First, each ecosystem service (pollution reduction, recreational activities, biodiversity and flood protection) has been identified by a specific pictogram. Second, the level of service provision by associated to a specific color (green for good level, yellow for medium level and red for bad level).

*Contingent valuation scenarios*. We have then proposed sequentially four different contingent valuation scenarios, again discussed and validated by IRIDRA and by the representatives of the Gorla Maggiore municipality. The four scenarios correspond to the exiting water park and to three alternative infrastructures that had been considered by the representatives of the Gorla Maggiore municipality. Respondents have been asked to evaluate these scenarios in comparison to the reference scenario (private poplar plantation). We have used the following script:

*“With respect to the reference situation you are asked to choose the best project to prevent the sewage from Gorla Maggiore to pollute the Olona River. To do so, you should value each one of the 4 projects proposed against the poplar plantation.”*

Each scenario has been obtained by combining a type of infrastructure dedicated to treat wastewater of the municipality of Gorla Maggiore (either a *green* or a *conventional* infrastructure) with the possibility to have or not a recreational park around this infrastructure (either a recreational *park* or a private *poplar* plantation). In the questionnaire the *green* and the *conventional* infrastructure where respectively defined as a set of constructed wetlands with a wet retention pond, and a flush tank (buried and covered by grass) with a dry retention pond. The recreational *park* was described as an area with trees designed for recreational activities whereas the private *poplar* plantation was presented as being non-accessible for recreational activities. By combining the type of infrastructure and the type of area surrounding, we get the four contingent valuation scenarios:

– P1: Green infrastructure & park;
– P2: Green infrastructure & poplar;
– P3: Conventional infrastructure & park;
– P4: Conventional infrastructure & poplar.

To make people more clearly understand the meaning of these four scenarios, each of them was described by two pictures (one for the infrastructure and another for the surrounding area). The pictures which have been shown to the respondents for each scenario are presented in Fig. 2.

The level of ecosystem services provided (in terms of pollution reduction, recreational activities, biodiversity and flood protection) associated to each scenario was also verbally and graphically presented. For the graphical representation, we use again some pictograms with a color representing the level of service provision (green for good level, yellow for medium level and red for bad level). For the verbal description, we have used the script presented in Fig. 2. It should be mentioned that the four scenarios allow to achieve a high level of pollution control (a mandatory requirement for the Gorla Maggiore municipality). However, the technical way to achieve pollution control significantly differs depending on the green or the conventional infrastructure. The provision of recreational services varies across scenarios from low in the scenario P4 (conventional & poplar) to high in the scenario P1 (green & park). The two other scenarios provide an intermediate level of recreational services. The level of biodiversity is assumed to be high in the scenario P1 and P2 (green & park and green & poplar) and low in the scenario P3 and P4 (conventional & park and conventional & poplar). Lastly, the four scenarios result in high flood control. Our approach is then conceptually similar to the attribute-based contingent valuation method proposed by Moore et al. (2011) in the context of forest protection.

*Hypothetical bias of the CV scenarios*. Hypothetical bias and consequentiality are a concern for any CV study. It may be an issue in our case since respondents have been asked to go back in time when considering the set of alternative infrastructures to be valued. In our setting we minimized the impact of the hypothetical bias using a cheap talk script:

*“Here below we present the 4 alternative projects to the reference situation and we ask for your personal valuation. For the following questions (No. 6–9), it is very important that you reflect your real intention. Imagine what the proposed reallocation of public budget means in terms of reduction of public good and services for your household (less money for public schools for example) and what types of benefits you will get from each project.”*

So we have put some emphasis on the need to provide personal valuation and to indicate real decision. There is evidence of the efficacy of cheap talk as a method for diluting the effects of hypothetical bias (Fifer et al., 2014, de Magistris and Pascucci, 2014) and some studies even suggest that the hypothetical bias can be totally eliminated by using an adapted cheap talk (Cummings and Taylor, 1999). In addition, it seems reasonable to think that the respondents were familiar with all the proposed options: the poplar plantation was the previous situation (until the construction of the green infrastructure in 2012), the traditional grey infrastructure is the common local solution present in the Lombardy region in most of municipalities, and the green infrastructure is the actual situation. Therefore, in the scenarios we have combined four elements that were equally known to the local people: the poplar, the park, the constructed wetland, the traditional retention basin. This local knowledge is also related to the fact that Gorla Maggiore is a small municipality in which the construction of the Gorla’s water park followed a highly participatory planning approach (Liquete et al., 2016).

*Format of the contingent valuation questions*. We wish to estimate the WTP for the 4 CV scenarios, in comparison to the reference scenario (poplar plantation). Although a willingness to accept (WTA) approach would have been a relevant alternative, we have preferred to elicit a WTP since it is known to be less affected by the hypothetical bias (Arrow et al., 1993).^1^

We have chosen to use a payment card (PC) approach, one of the most popular method for eliciting WTP in environmental valuation where respondents are presented with a set of ordered payment amounts, or bids, and typically are asked to circle the maximum of the series they would pay for the good under valuation. The PC method was first developed by Mitchell (1981). The main advantages and disadvantages of the PC format as opposed to other methods are fully discussed in Mitchell and Carson (2013) and some specific examples of empirical comparisons between WTP through PC and through other formats include (Blaine et al., 2005). In our case, the bid structure was constructed based on experts’ suggestions and based on the actual construction and maintenance costs of the Gorla Maggiore park. It covers a range going from zero euro per household and per year to more than 75 euros per household and per year.

The choice for the payment vehicle is a crucial element for any contingent valuation survey since it provides the context for payment Morrison and Blamey (2000). Our pre-tests and the discussions we have had with the representatives of Gorla Maggiore municipality leaded to the conclusion that using a tax increase for funding the infrastructure could not be considered in the current economic and political context in Italy. Hence, due to the economic crisis, a lot of people may be per principle opposed to any taxation increase. We have then decided to use the municipality budget (which is funded in Italy through local taxes) as a payment vehicle making explicit that any money dedicated to fund the proposed infrastructure would then not be available for funding the provision of other municipal public goods. Although we recognize that this payment vehicle is not fully satisfactory from an incentive point of view, it is the second-best option in our setting. The script used for explicating the payment vehicle is presented in Fig. 2. This figure also gives PC questions used for the different contingent valuation scenarios.

### 3.3 Survey administration and sampling issues

The mode of administration for CV surveys has been highly debated in environmental economics (Lindhjem and Navrud, 2011). Mitchell and Carson (2013) have argued that the preferred mode of administration for CV surveys is in–person interviews conducted in the respondent’s home. The main rational is the need to explain complex scenarios using visual aids with control over pace and sequence. Mitchell and Carson (2013) have however acknowledged that mail survey may be suitable for surveying respondents who have familiarity with the good (e.g. recreational users). This is typically the case here. As a result, the survey has been distributed by mail to all households living in the municipality of Gorla Maggiore beginning of 2015. The questionnaire has been included into the newsletter regularly sent by the municipality to all households, and it has been directly advertised on the web site of the Gorla Maggiore municipality. Then, households were given the choice either to directly fill in the questionnaire and to put it back into a dedicated urn at the townhall of the municipality, or to fill the questionnaire online on a dedicated web site (EU-survey).

In total, 1600 questionnaires have been distributed to households living in Gorla Maggiore. We have received 71 full questionnaires (25 from EU-survey, and the remaining from the dedicated urn at the townhall of the municipality). This translates to a low response rate (4.4%) which is not surprising given the Italian economic and political context and the fact that we have used a mail survey. This raises however some issues related to the representativeness of our sample we discuss below.

A few papers have questioned the use of survey data in case of low response rates (Keeter et al., 2006, Smith, 2009, Rindfuss et al., 2015). A consensus which emerges from these works is that a low response rate does not necessarily lead to biased results. For example, Smith (2009) conducted a study in the US with a mail-out mail-back survey. After obtaining an initial low response rate, he selected a small sub-sample of non-respondents, and used financial incentives to improve response rate. Comparing the low and high-response surveys, Smith (2009) reports no evidence of bias in the low-response survey.

In Table 1 we compare some selected socioeconomic characteristics of our respondent sample with the characteristics of the population living in Gorla Maggiore, in Lombardy and in Italy. On average the household size in our sample is higher than what is reported by the Italian National Institute for Statistics (ISTAT) for the municipality of Gorla Maggiore in 2014 (2.86 versus 2.45 persons per household). With 38.0% only, females are under-represented in our sample. On average, our respondents are slightly older than inhabitants in Gorla Maggiore. The average annual household income in our sample is 30,794 euros. This amount is in between what is reported for Italy (29,436 euros) and for Lombardy (34,097 euros). Lastly the percentage of respondents considered as economically active (i.e currently employed and unemployed) in our sample matches very well the data reported by ISTAT for Gorla Maggiore in 2011. Although we do not claim that our sample is representative of the population living in the municipality of Gorla Maggiore, the previous analysis suggests no indication of strong differences with ISTAT data for the municipality of Gorla Maggiore based on the observable characteristics presented in Table 1, with the exception of the share of females.

**Table 1:** Socio-economic characteristics of the respondent sample.

Variable	Italy	Lombardy	Gorla Maggiore	Our sample
Household size (in 2014)	2.34	2.26	2.45	2.86
Female (in 2014)	51.5%	51.2%	50.2%	38.0%
Average age population above 18 (in 2014)	51.1	51.3	51.5	54.8
Household annual income (in 2012)	29,436	34,097	29,120⁎	30,794
Population economically active (in 2011)	50.8%	54.8%	53.8%	53.5%

⁎ For municipalities in Lombardy with less than 2,000 inhabitants Socio-economic data for Italy, Lombardy and Gorla Maggiore come from ISTAT.

## 4 Empirical results from the contingent valuation survey

### 4.1 Use and perception of the Gorla Maggiore park

The first part of the questionnaire has been dedicated to collect data related to the way the Gorla Maggiore park is used and perceived by the respondents. On average, each respondent has visited the park a little bit more than 25 times over the last 12 months (SD is 31.89). In our sample, the annual number of visits varies from 0 (for 5 respondents) to more than twice a week (for 7 respondents). The average typically size of the group when the respondent goes to the park is 2.43 (SD is 1.27), varying from 1 (for 14 respondents) to more than 5 (for 4 respondents). Respondents typically live in the proximity of the park. The average distance to the park is 1.38 km (SD is 0.74). For 27 respondents the distance to the park is less than 1 km.

Next, each respondent has been proposed a list of 8 possible recreational activities, see Table 2. Each respondent has then been asked how often he has practiced each activity in the last 12 months. Sightseeing and walking/ dog walking are by far the two types of recreational activity which are the most often undertaken by park visitors. 36 respondents have declared that they go to the park at least time to time for running or biking, or for watching wildlife. Educational or leisure activities with kids are also mentioned by some respondents. The main insight we get from Table 2 is that the Gorla Maggiore is used for wide range of recreational activities.

**Table 2:** Frequency of recreational activities in the Gorla Maggiore Park.

Activity	Never	Sometimes	Often	Sometimes or Often
Walking or dog walking	5	16	36	52
Running or biking	10	19	17	36
Educating children to nature	18	11	8	19
Playing with kids	19	12	5	17
Picnicking	30	4	0	4
Watching wildlife (birds/frogs)	8	18	18	36
Sightseeing/enjoying nature	1	22	32	54
Sunbathing	27	9	1	10

Number of respondents having practiced a given activity in the last 12 months.

### 4.2 Preliminary analysis of answers to contingent valuation scenarios

Now we move to the answers given by the respondents to the four contingent valuation scenarios P1, P2, P3 and P4 described above.

Table 3 gives some statistics on the maximum amount of money each respondent is ready to allocate to each contingent valuation scenario (in euro per year and per household for the following twenty years). We interpret this amount of money as a WTP for the corresponding scenario.

**Table 3:** Descriptive Statistics on WTP per contingent valuation scenario.

	Mean	Std. Dev	Min	Max	Observations
*Full sample*					
P1: green infrastructure & park	26.20	20.45	0	75	71
P2: green infrastructure & poplar	9.28	12.13	0	45	58
P3: conventional infrastructure & park	5.39	12.46	0	75	61
P4: conventional infrastructure & poplar	3.20	10.28	0	75	61

*Sample without false zeros*					
P1: green infrastructure & park	28.19	19.83	0	75	66
P2: green infrastructure & poplar	10.15	12.34	0	45	53
P3: conventional infrastructure & park	5.88	12.90	0	75	56
P4: conventional infrastructure & poplar	3.48	10.69	0	75	56

Willingness to pay in euro per year and per household.

In a contingent valuation analysis, it is important to make the distinction between the “true zero bids” corresponding to respondents having indicated that they are not willing to pay anything because they are truly averse or indifferent to the good for which a WTP is solicited from “false zero bids” which correspond to respondents having reported a zero WTP even though her true value for the good in question is positive, Hanley et al. (2006). False zero bids may be categorized into three types. The first are “protest bids”, where the respondent reports a zero bid for reasons other than the respondent placing a zero value on the good in question. The second are “do not know” responses, where the respondent is simply uncertain about the amount they are willing-to-pay, noting that this amount could of course be zero. Third, some respondents may have stated a zero bid because the task of selecting options is too complex (i.e., they have difficulties understanding or answering the choice questions).

To identify protest answers, respondents having reported zero WTP for the four proposed scenarios have been asked if they agree or disagree with the six following reasons: “1- I am not confident that the money will be used efficiently by the municipality”, “2- I am against any tax expenses”, “3- I prefer the money to be spent on more important things”, “4- I cannot afford to pay any tax”, “5- I believe that the park should not be paid by me but directly by a central administration” and “6- I will never go to the park”. All respondents have also been asked to state if the survey was clear, which is the case for 95.6% of respondents. Among the 6 respondents having reported zero WTP for the four proposed scenarios, 5 who have selected at least one of the reason 1-, 2- or 5-, can be classified as “false zeros”. In Table 3 we then report some statistics on WTP per scenario first based on the full sample and second on a subsample excluding “false zeros”.

Table 3 calls for a few comments. First, whatever the sample considered there are significant differences across the WTP per scenario which varies from around 3 euros per household and per year for scenario P4 (conventional infrastructure with poplar plantation) to 26 to 28 euros for P1 (green infrastructure with park). Second, for a given surrounding area respondents have a higher WTP if the infrastructure is green compared to the conventional one. Considering the sample without “false zeros”, passing from a conventional to a green infrastructure increases the WTP by 6.67 euros per respondent and per year for a surrounding made of poplars and by 22.31 euros per respondent and per year for a surrounding made of a park. Third, compared to the three other scenarios, we find a much higher WTP for P1 which corresponds to the green infrastructure with park (the one which has been built in the Municipality of Gorla Maggiore). This may be related to the specific attributes of P1 but it may also be the result of a strong “endowment effect” since P1 is the infrastructure which has been really built. The “endowment effect” refers to the theory that explains observed gaps between WTP and willingness to accept (WTA) by some feature of human preferences that leads owners to resist selling goods because (a) selling is perceived as “losing” the endowed good, and (b) individuals are generally loss averse Plott and Zeiler (2005). The “endowment effect” has been highly documented in contingent valuation studies, see Tuncel and Hammitt (2014). One should lastly point out that there may be some other explanations for the higher WTP attributed by respondents to P1. These possible explanations include the presence of an income effect, of transaction costs, the absence of credible substitutes to the existing park and the limited incentives to learn about preferences for a hypothetical transaction.

To gain some insights on how WTP differs across individuals, we provide in Table 4 the WTP for scenario P1 (green infrastructure & park) for several subsamples.

**Table 4:** WTP for scenario P1 (green infrastructure & park) by subsample.

	Mean	Std. Dev	Min	Max	Observations
*Number of visits per year*					
– None	21.40	19.93	0	45	5
– [1, 20]	24.54	15.12	0	62.5	38
– >20	35.70	24.75	0	75	23

*Distance to the park (in km)*					
– ⩽1	27.14	19.30	0	75	25
–]1, 2]	28.46	20.20	0	75	32
– >2	30.11	22.11	0	75	9

*Level of appreciation of the park*					
– Low	24.80	31.15	2	75	5
– Medium	27.01	15.89	0	62.5	30
– High	30.40	21.59	0	75	29

*Age of respondent (in years)*					
– ⩽40	35.28	23.34	2	75	16
–]40,50]	34.03	17.02	10	75	15
– >50	22.44	17.88	0	62.5	35

*Household income (in euros per year)*					
– ⩽5,000	16.40	18.38	0	45	15
–]15,000 to 30,000]	31.14	19.95	2	75	29
– >30,000	32.34	18.22	0	75	22

*Sex of respondent*					
– Female	28.56	21.93	0	75	24
– Male	27.98	18.80	0	75	42

Willingness to pay in euro per year and per household, false zeros excluded.

As expected, the WTP increases with number of visits to the park during the last 12 months, from 21.40 for respondents reporting no visits to 35.70 euros for those having visited the park more than 20 times. The WTP for respondents located less than 1 km from the park and for those located more than 2 km from the park are not statistically different at 5%. The WTP does not appear to vary with the distance to the park. Respondents who have a low appreciation of the overall quality of the Gorla Maggiore Park report a low WTP (24.80 euros) but they represent only a small fraction of the sample (5 respondents).

Concerning the socioeconomic characteristics, we find a significantly lower WTP for oldest respondents. The average WTP for respondents over 50 years is only 22.44 euros per year. One should however be careful with interpreting this result as a pure age effect since oldest respondents may have some specifics characteristics affecting their WTP (i.e. low income or low frequency of park visit). We find a significant income effect, especially for the poorest respondents. The average WTP for households reporting an annual income lower than 15,000 euros is only 16.40 euros per household. It is approximatively equal to half of the WTP reported by wealthier households. Lastly, our results do not reveal any significant difference between female and male WTP. This result is important since, as discussed previously, females are under-represented in our sample. Since sex does not matter, we do not anticipate that the under-representation of females will affect our final estimates of the WTP.

### 4.3 Econometric analysis of WTP

When analysing data obtained from a PC contingent valuation survey, it is unclear what assumptions should be made regarding respondent’s true WTP. A standard approach is to assume that the WTP follows a normal distribution. The valuation function can then be represented by:

(1) $${\mathrm{WTP}}_{i}^{*}=X_{i}^{\prime }\beta +{∊}_{i}$$

where ${\mathrm{WTP}}_{i}^{*}$ denotes the true WTP for respondent $i\text{,}X_{i}$ a vector of explanatory variables and ${∊}_{i}$ a random component following a normal distribution with mean zero and standard deviation *σ*.

A standard procedure to estimate Eq. (1) is to assume that the true WTP is the midpoint between the highest amount to which the respondent said “yes” and the lowest amount to which she said “no” Cameron (1987). This approach allows direct estimation of WTP, thus no assumptions are made regarding the functional form of respondents’ utility or the error structure of the data. A straightforward analysis consists then in simply regressing the stated WTP on various explanatory factors but Cameron, 1987, Cameron and Huppert, 1989 have showed however that this type of analysis is generally not efficient.

An alternative is to explicitly consider the structure of data obtained from a PC contingent valuation survey. Since respondents are asked to select the maximum amount of money they would pay for the good under valuation, it means that the individual’s WTP is bounded by the largest amount they agreed to pay and the smallest amount they refused (interval censoring). If the highest payment is chosen, the WTP is assumed to be located somewhere above this payment (right–censoring). If the lowest payment is chosen, the WTP is supposed to be below this payment (left–censoring). The usual parametric approach to estimate the valuation function with censored data in the dependent variable is the “interval data model” Cameron and Huppert (1989). When considering the interval data model, the contribution of each response to the likelihood function is given by the probability that the latent WTP value falls within the chosen interval. This probability is then found by taking the integral of the conditional probability density function over the range of WTP indicated by the interval response, but the specific form for the probability depends upon the type of censoring in the interval data model (interval censoring, right–censoring or left–censoring). Interval censoring corresponds to the case where ${\mathrm{WTP}}^{*}$ lies in the bracket bounded by the payment chosen and the next amount in proposed list denoted $t_{\mathrm{li}}$ and $t_{\mathrm{ui}}$. In the right–censoring case, ${\mathrm{WTP}}^{*}$ is greater than $t_{\mathrm{li}}$ whereas the left–censoring case correspond to a ${\mathrm{WTP}}^{*}$ lower than $t_{\mathrm{ui}}$. The conditional probability of observing each case for respondent *i* writes:

(2) $$P({\mathrm{WTP}}_{i}^{*}|X_{i})=\left\{\begin{array}{cc} \Phi \left(\frac{t_{\mathrm{ui}}-X_{i}^{\prime }\beta }{\sigma }\right)-\Phi \left(\frac{t_{\mathrm{li}}-X_{i}^{\prime }\beta }{\sigma }\right) & \;\text{if interval-censoring}\\ 1-\Phi \left(\frac{t_{\mathrm{li}}-X_{i}^{\prime }\beta }{\sigma }\right) & \;\text{if right-censoring}\\ \Phi \left(\frac{t_{\mathrm{ui}}-X_{i}^{\prime }\beta }{\sigma }\right) & \;\text{if left-censoring} \end{array}\right)$$

where Φ is the cumulative standard normal density function. The corresponding log–likelihood function is made of three parts, which correspond to interval–censoring, left–censoring and right–censoring observations.

Since each respondent is asking to answer several CV questions, our approach requires further generalization of the model presented above. Multiple responses per individual are likely to induce some degree of correlation within responses Moore et al. (2011). To control for potential intra-individual correlation, we used a random effects panel model, which assumes that intra–individual correlation is randomly distributed over the sampled population. A random effects model with normally distributed errors and latency in the dependent variable yields

(3) $${\mathrm{WTP}}_{\mathrm{ij}}^{*}=X_{\mathrm{ij}}^{\prime }\beta +u_{i}+{∊}_{\mathrm{ij}}$$

with $u_{i}$ follows a normal distribution with mean zero and standard deviation $\sigma_{u}$ and ${∊}_{i}j$ follows a normal distribution with mean zero and standard deviation $\sigma_{∊}$. In Eq. (3), ${\mathrm{WTP}}_{\mathrm{ij}}^{*}$ is the latent value known to individual *i* in response to the *j*th question but unobserved by the researcher, $X_{\mathrm{ij}}$ is a vector of the data for that response, and *β* is a vector of coefficients. In the random effects model the error is decomposed into two components. The term $u_{i}$ is a random error that varies across individuals but is constant within an individual’s set of responses. The term ${∊}_{\mathrm{ij}}$ is a random error that can vary across individuals and responses. The two error components, $u_{i}$ and ${∊}_{\mathrm{ij}}$, are assumed to be independent and identically distributed and independent of each other. The conditional probability of observing a sequence of choice for individual *i* for all CV questions is obtained from Eq. (2) by multiplying the corresponding probabilities. The model has been estimated using the random effects interval data model (xtintreg) with the Stata statistical package.

We present in Table 5 some random–effects regression models. Model 1 only includes the type of infrastructure valued. Model 2 includes in addition some socioeconomic characteristics of respondents. The two first columns correspond to the full sample whereas in columns 3 and 4 the false zeros have been excluded.

**Table 5:** Random–effects regression models.

	Full sample		Sample without false zeros	
	M1	M2	M1	M2
Green infrastructure (0/1)	6.30^***^	6.59^***^	6.88^***^	7.11^***^
	(1.82)	(1.87)	(1.89)	(1.92)
Park (0/1)	2.17	2.28	2.36	2.44
	(1.79)	(1.84)	(1.85)	(1.89)
Green infrastructure & Park (0/1)	14.72^***^	15.52^***^	15.91^***^	16.47^***^
	(2.53)	(2.60)	(2.62)	(2.67)
Dummy if children below 18 (0/1)		−0.41		−0.73
		(2.74)		(2.73)
Dummy respondent age over 50 (0/1)		−2.94		−2.10
		(2.56)		(2.59)
Dummy for annual number visit >20 (0/1)		6.73^***^		7.84^***^
		(2.43)		(2.50)
ln household annual income (euros)		6.83^***^		6.23^***^
		(2.33)		(2.39)
Constant	2.58	−68.20^***^	2.64	−62.76^**^
	(1.68)	(24.16)	(1.73)	(24.74)
$\sigma_{u}$				
Constant	8.82^***^	7.63^***^	8.76^***^	7.59^***^
	(1.10)	(1.04)	(1.14)	(1.06)
$\sigma_{e}$				
Constant	9.77^***^	9.79^***^	9.68^***^	9.67^***^
	(0.57)	(0.45)	(0.56)	(0.48)

Log likelihood	−737.59	−691.18	−657.67	−626.92
N. of obs.	249	237	229	221

Estimated coefficients and standard errors in parentheses.

^***^,^**^,^*^ respectively for significant at 1, 5 and 10%.

Three dummy variables have been introduced for describing the scenario under consideration. Green infrastructure is a dummy variable equal to 1 if the infrastructure considered is green (the reference category is a conventional infrastructure). Park is a dummy variable equal to 1 if the surrounding area is a recreational park (the reference category is a private poplar plantation). Since the previous analysis has suggested that there might be a premium for the scenario combining the green infrastructure and a recreational park, a third dummy variable has been added to account for this situation. As explanatory variables, we have introduced a dummy variable for respondents indicating that there is at least one child below 18 years in their household and another dummy variable equal to 1 if the respondent is over 50 years old. Household income is introduced in logarithm and we also control for the frequency of visits to the park.

Table 5 calls for some remarks. First, both the sign and the order of magnitude of the estimated coefficients are very similar across models. Concerning the characteristics of the scenarios under study, we find a positive and significant WTP for the green infrastructure (compared to the conventional one). Depending upon the model considered the WTP for a green infrastructure varies from 6.3 to 7.1 euros per household and per year (for a twenty years time horizon). We also find a positive (but not significant) WTP for a park varying from 2.2 to 2.4 euros per household and per year. The most interesting finding is given by the positive and highly significant coefficient for the interaction between the green infrastructure and the park. There is a specific premium for a project combining a green infrastructure together with a recreational park. This premium is quite significant in terms of amount of money since it varies from 14.7 to 16.5 euros per household and per year, depending upon the model considered. Our results suggest that people in Gorla Maggiore do not put any specific value on a park if it associated with a conventional infrastructure. On contrary the park will be highly valued if is associated with the green infrastructure. One possible interpretation of this result is that the park and the green infrastructure may be perceived as two highly complementary goods. Another explanation is the “endowment effect” we have discussed previously.

As expected from the descriptive statistics, WTP is significantly impacted by respondent’s income and respondent’s frequency of visits to the Gorla Maggiore park. The higher is the household income, the higher will be the WTP. In addition, respondents reporting that they went to the park at least 20 times during the last 12 months have an additional WTP which varies between 6.7 and 7.8 euros per household and per year.

Since the four alternative infrastructures are directly related to the level of ecosystem services they provide (attributes “low”, “medium” and “high”), our estimates may directly be interpreted in terms of WTP per attribute. More specifically, the WTP for high and medium levels of recreational activities is estimated to be 19.04 and 2.16 euros per household and per year (reference category is low level of recreational activities). The WTP for a high level of biodiversity is estimated to be 4.13 euros per household and per year (reference category is low level of biodiversity). Finally the joint WTP for a high level of pollution control and a high level of flood control is estimated to be 2.57 euros per household and per year.

### 4.4 Using contingent valuation for informing public decision-making

We perform in this section some back-of-the-envelope calculation to provide an estimate of the net benefits resulting from the implementation of the four contingent valuation scenarios. We use a cost-benefit analysis (CBA) approach to compare the relevance of the proposed alternative infrastructures based on a monetary criterion. This retrospective analysis provides a way for policy-makers to check if the decision to build the Gorla Maggiore water park can be rationalized ex-post based on some economic criteria. A more comprehensive approach would have been to incorporate the costs and benefits of the co-production process into an ecosystem services accounting, but the methodology is still under development (Villa et al., 2014).

Implementing a CBA implies to compare some costs and benefits that may occur at different dates. This is particular important in our case since each of the four proposed infrastructures has a life expectancy of 20 years. To make these monetary flows comparable, costs and benefits must then be expressed in present terms. This raises the issue of using an appropriate discounting rate. As well-known, net present values are highly sensitive to the choice of the discount factor, especially when there is some uncertainty regarding the values to be discounted Gollier and Weitzman (2010). When conducting our CBA, we will then report the discounted net benefits for each scenario for three different interest rates (2%, 3% and 4%).

To compute the total cost of each infrastructure, we have relied on information provided by IRIDRA, the engineering private firm which was responsible for the design and the construction of the Gorla Maggiore park. We have considered both construction and maintenance costs. When presenting these costs in Table 6, we make the distinction between infrastructure and landscaping expenses since they differ across the proposed infrastructures.

**Table 6:** Cost benefit analysis of contingent valuation scenarios.

	Construction cost		Maintenance cost		IndividualWTP	Discounted net benefits†		
	1000 €		1000 € per year		€ per year	1000 €		
						Interest rate		
Scenario	(a)	(b)	(a)	(b)		2%	3%	4%
*Political jurisdiction market extent: Gorla Maggiore municipality (2,045 households)*								
P1: green & park	820.0	80.0	2.6	1.0	28.2	5	−68	−132
P2: green & poplar	820.0	0.0	2.6	0.0	10.2	−515	−539	−561
P3: conventional & park	794.7	50.0	11.8	3.6	5.9	−886	−881	−877
P4: conventional & poplar	794.7	0.0	11.8	0.0	3.5	−861	−855	−849
*Economic jurisdiction market extent: Gorla Maggiore and Fagnano Olona municipalities (6,907 households)*								
P1: green & park	820.0	80.0	2.6	1.0	28.2	2,292	2,033	1,805
P2: green & poplar	820.0	0.0	2.6	0.0	10.2	308	217	137
P3: conventional & park	794.7	50.0	11.8	3.6	5.9	−409	−443	−473
P4: conventional & poplar	794.7	0.0	11.8	0.0	3.5	−579	−595	−610

(a): Infrastructure.

(b): Landscaping.

† Aggregated net benefits discounted over a period of 20 years.

The measure of the benefits is less straightforward, first because our WTP may not cover the full range of services offered by the park, and second due to the need to define the relevant market on which individual benefits must be aggregated.

In our CV setting, we have considered the four main ecosystem services delivered by the park (i.e. pollution control, flood prevention, recreational use and biodiversity or wildlife support). Although these services have been recognised to be of first importance by stakeholders, the Gorla Maggiore park may deliver additional services which will not be accounted for here. This is for example the case for the educational service (the park is used by local primary schools for teaching ecology to pupils) or for the local climate regulation service (the park contributes to micro and regional climate regulation and to air quality). It follows that our benefit measure should be viewed as a lower bound of the true value of the proposed infrastructures.

The relevant market (i.e the area on which individual benefits are aggregated) must be first defined. This market delineation is known to be one of the most controversial issue in environmental valuation (Bateman et al., 2006). We consider two extents of market respectively based on a *political jurisdiction* and an *economic jurisdiction* approach. A political jurisdiction is a conservative definition of the market extent limited to households sharing the cost of implementing the proposed infrastructure (Pate and Loomis, 1997, Bateman et al., 2006). In our case, the political jurisdiction corresponds to households belonging to the Gorla Maggiore municipality where the park has been built.

An economic jurisdiction is an alternative definition of the market extend which consists in accounting for all households who hold economic values regarding the proposed infrastructure (Bateman et al., 2006). In our specific case, the Gorla Maggiore proposed infrastructures forms the border between two municipalities namely Gorla Maggiore and Fagnano Olona, the later one having also a direct access to the park. We will then consider an economic jurisdiction corresponding to all households living in the municipalities of Gorla Maggiore and Fagnano Olona. It is clear that other definitions for the market extent could have been considered, especially since all beneficiaries from the services provided by the proposed infrastructures may not necessarily belong to the political or the economic jurisdictions. For instance, the regulating services such as pollution and flood control will benefit in the first place to households in the municipalities of Gorla Maggiore and Fagnano Olona, but also to households in municipalities located downstream. A larger market may then be considered for the aggregation of individual benefits. The aggregated benefits for a given valuation scenario are then given by multiplying the individual WTP reported in Table 6 by the number of households belonging to the relevant market. We implicitly assume that the WTP is not impacted by the distance to the proposed infrastructure. The interested reader may refer to Bateman et al., 2006, Kozak et al., 2011, Schaafsma et al., 2012, Sen et al., 2014, Perino et al. (2014) for works having considered spatial decay functions in the context of environmental valuation studies. Since the relevance of using a distance decay at a very local scale has never been empirically validated, we do not consider this issue in the spatial aggregation of benefits.

Results presented in Table 6 call for a few comments. First, the definition of the market extent matters for the result of the CBA. With a *political jurisdiction* definition of the market, we get a positive net present value only for the scenario P1 (green infrastructure & park) whereas by considering an *economic jurisdiction* definition, both scenario P1 (green infrastructure & park) and P2 (green infrastructure & poplar) result in a positive discounted net benefit. Second, the CBA results are also highly impacted by the choice of the discount factor. For instance, when considering an interest rate equal to 2% with a *political jurisdiction* definition of the market, we get a positive discounted net benefit equal to 5121 euros. The discounted net benefit becomes negative with a 3% interest rate. Third, whatever the interest rate considered, scenario P1 (green infrastructure & park) provides the highest discounted net benefits. This is not surprising given the high individual WTP for this infrastructure. Fourth, whatever the interest rate and the market extent definition, the net present value of benefits for scenario P3 (conventional infrastructure & park) and P4 (conventional infrastructure & poplar) are always negative, which means that they should not be implemented based on our CBA criterion. This result may be driven by the rather restrictive definition of the market extent we have used in Table 6, and by the benefits we have accounted for. Indeed it should be stressed that by relying on a WTP approach, we have not formally measured the total social benefits associated to each of the four proposed infrastructures, but mainly an associated direct use value. Inclusion of non-use values and values related to potential future use (option and bequest values) may have significant impacts on the result of the CBA (Hanley et al., 2003). In addition, some indirect effects of building a park such as enhancement of community cohesion or increase in nearby residential property values are not accounted for in our analysis.

## 5 Conclusion

A contingent valuation approach has been used to estimate how households value different multipurpose infrastructures (conventional or green) for managing water pollution and flood control. As a case study we have considered the Gorla Maggiore water park located in the Lombardy Region, in Northern Italy. This neo-ecosystem which includes a green infrastructure to treat waste water, store excess rain water and provide recreational services to the population, is the first one of this type built in Italy. A novel aspect of our research is that it not only considers the values people hold for different water ecosystem services (pollution removal, recreative use, biodiversity, flood risk reduction), but also their preferences for how those outcomes are achieved (through conventional or green infrastructures). To this end, we have implemented an attribute-based contingent valuation approach Moore et al. (2011). The results indicate that the type of infrastructure delivering the ecosystem services (conventional or green) does have an impact on individuals’ preferences for freshwater ecosystem services. By considering the type of infrastructures within the choice model, we gain a richer understanding of the relationship between social welfare and freshwater ecosystem services.

Our empirical results reveal a positive and significant WTP for the green infrastructure (compared to the conventional one). Moreover, we find a specific premium for a project combining a green infrastructure together with a recreational park. This premium is quite significant since it varies from 14.7 to 16.5 euros per household and per year, depending upon the model considered. The WTP depends on some characteristics of respondents. In particular, it is significantly impacted by respondent’s income and respondent’s frequency of visits to the Gorla Maggiore park.

We argue that WTP surveys may be useful for regional planning Vandermeulen et al. (2011). As demonstrated in our paper, the elicited WTP may help decision-makers to prioritise their long-term investment decisions. In addition, the survey can be an important instrument of stakeholder participation in regional spatial planning Wilker and Rusche (2014). In our case, both the representatives of the Gorla Maggiore municipality and the Lombardy region have been directly involved into the design of the survey and the analysis of the results. We believe that both a good understanding of the benefits local populations get for green infrastructures and involvement of local stakeholders in the decision-process are two important components of any welfare-enhancing regional spatial planning. From a policy perspective, we also believe that implementing our contingent valuation survey in municipalities which are considering the possibility to build similar green infrastructures in Lombardy could provide complementary results to the ones presented here.

Lastly, even if urban parks may be viewed as a cost-effective solution for providing multiple ecosystem services, their development at a large scale may raise some policy challenges. First, green open spaces usually benefit to a population dispersed on a wider area than the one actually supporting the cost of the infrastructure (the political and the economic jurisdictions usually do not fully overlap). This may result in a free riding problem and an under-provision of this kind of public good. Second, in some urban areas building a green infrastructure may create a tension between the high value of land for development and the greater demand for these spaces due to the high numbers of people. Again, involvement of local stakeholders in the decision-process emerges as a crucial issue.

## Footnotes

1 Choosing between eliciting WTP and WTA has been highly debated in the academic literature, and existing meta-analyses on hypothetical bias in stated preference reveal significant differences between these two approaches (List and Gallet, 2001, Little and Berrens, 2004).
